# School bullying and non-suicidal self-injury among rural adolescents: the mediating role of alexithymia and the moderating role of friendship quality

**DOI:** 10.3389/fpsyg.2025.1596166

**Published:** 2025-09-09

**Authors:** Jing Wen, Qinghong Xu, Hongjun Zhang, Jianli Ding, Min Li

**Affiliations:** ^1^School of Education, Inner Mongolia Minzu University, Tongliao, China; ^2^Inner Mongolia Student Bullying Prevention Research Center, Tongliao, China; ^3^Inner Mongolia Ethnic Education and Psychological Development Research Base, Tongliao, China; ^4^School of Foreign Languages, Yulin University, Yulin, China

**Keywords:** student bullying, non-suicidal self-injury, alexithymia, friendship quality, the risk-buffering model

## Abstract

This study investigates the relationship between school bullying and non-suicidal self-injury (NSSI) in adolescents, with a particular focus on the mediating role of alexithymia and the moderating effect of friendship quality. The present study employed the Bullying Questionnaire, the Toronto Alexithymia Scale, the Friendship Quality Questionnaire, and the Adolescent Non-Suicidal Self-Injury Assessment Questionnaire with 701 middle school students. The results indicate that (1) Student bullying significantly predicts NSSI (79.92%) and (2) alexithymia partially mediated this relationship (20.08%). Moreover, (3) friendship quality was identified as a significant moderator in the relationship between student bullying and NSSI (*β* = −0.07, *p* < 0.05). Furthermore, friendship quality also moderated the relationship between alexithymia and NSSI (β = −0.003, *p* < 0.01). In conclusion, the study highlights the mediating role of alexithymia and the moderating role of friendship quality, providing valuable insights for psychological interventions targeting adolescent populations and adding to the social buffer theory. Friendship quality may reduce non-suicidal self-injury and alleviate the symptoms of alexithymia, which explores friendship quality as a protective factor for alexithymia.

## 1 Introduction

Non-suicidal self-injury (NSSI) refers to the deliberate infliction of harm on one’s own body without the intent of suicide (Ahn et al., 2021). Adolescents, in particular, are highly susceptible to engaging in NSSI, with the behavior typically peaking between 15 and 17 years of age. The prevalence of NSSI among adolescents has been increasing, with studies showing a clear upward trend (Plener et al., 2015). In China, the reported prevalence of NSSI among adolescents ranges from 7.5 to 46.5% (Zhou and Jiang, 2021). Crucially, NSSI is a strong predictor of suicidal behavior (Asarnow et al., 2011; Tang et al., 2011; Wilkinson et al., 2011). The rising suicide rate poses a significant challenge to social stability and development. When adolescents view NSSI as a coping mechanism for stress, they may be more likely to engage in the behavior repeatedly, thereby reinforcing a harmful cycle (Joiner, 2005). Therefore, understanding the underlying mechanisms of NSSI is essential for developing effective interventions to mitigate suicide risk.

In recent years, research on the relationship between bullying and adolescent NSSI has significantly increased. Experiences of bullying constitute a significant risk factor for the development of NSSI (Ayano et al., 2021). Bullying is typically defined as a deliberate, aggressive act by a more powerful individual directed at a weaker one, characterized by repetition, an imbalance of power, and intentional harm. According to general stress theory, individuals exposed to stressors may engage in maladaptive behaviors to alleviate the emotional distress caused by these stressors (Van Geel et al., 2015). Given that bullying is widely regarded as a significant stressor (Hay et al., 2010), it can hinder an individual’s ability to cope with stress, leading to negative emotional responses such as anxiety, depression, and other psychological distress (Eastman et al., 2018; Ye et al., 2023). As a result, adolescents may turn to NSSI as a means of managing their emotional discomfort.

The detrimental effects of bullying experiences on adolescents are far-reaching, with alexithymia emerging as one of its notable consequences. Alexithymia is an impaired ability to recognize, process, and regulate emotions, typically manifested in difficulties identifying and describing feelings, distinguishing emotions from physical sensations, and a lack of imaginative or outward-directed thinking (Taylor, 2000). Research has identified a strong positive correlation between bullying experiences and increased levels of alexithymia. For example, Levantini et al. (2023) found that individuals who had been bullied scored significantly higher on alexithymia measures than those who had not (Levantini et al., 2023). Furthermore, Beck’s cognitive model of emotional disorders suggests that adverse life events can lead to the development of unconscious negative thought patterns. These cognitive biases can perpetuate or intensify maladaptive behaviors and emotional distress (Beck, 1976). Consequently, persistent bullying may reinforce negative self-schemas, impairing emotional awareness and expression—core features of alexithymia. Then, for individuals with emerging or existing alexithymia, emotional dysregulation stemming from unexpressed distress may increase vulnerability to NSSI as a maladaptive coping mechanism.

Moreover, research exploring the relationship between alexithymia and NSSI indicates that difficulties in identifying and articulating emotions may increase the likelihood of NSSI (Norman and Borrill, 2015). The cognitive-emotional model of NSSI suggests that individuals who struggle with emotional identification and expression, coupled with deficits in emotional regulation, are more likely to adopt NSSI as a coping strategy. NSSI often emerges as a coping mechanism for emotional distress caused by both external stimuli and internal emotional responses (Hasking, 2016). These findings lend strong theoretical support for the mediating role of alexithymia in the relationship between bullying victimization and NSSI.

Several studies have consistently demonstrated that alexithymia mediates the relationship between victimization and severe psychological outcomes. For instance, early research by Guzzo et al. (2014) highlighted that alexithymia mediates the link between bullying victimization and the development of post-traumatic symptoms. Their study found that difficulties in identifying and describing emotions, along with a tendency toward external thinking, play a significant role in linking bullying victimization to the onset of post-traumatic symptoms in adolescents (Guzzo et al., 2014). More recent studies explored how sociocultural and environmental factors influence alexithymia (Larsson et al., 2010; Taylor et al., 1991). From the social mechanism of alexithymia, adverse social factors during childhood can hinder socialization, impair emotional regulation, and contribute to maladaptive behaviors (Honkalampi et al., 2004). This framework underscores the importance of external social factors in developing alexithymia. Bullying, as a form of social trauma, disrupts the ability to identify and express emotions effectively. When negative emotions are poorly regulated, individuals may resort to NSSI as a way out. Based on these findings, the present study hypothesizes that alexithymia mediates the relationship between students’ experiences of bullying and NSSI.

Previous studies have demonstrated that emotional dysregulation and rumination serve as sequential mediators between experiences of school bullying and NSSI. Building on this understanding, the present study investigates whether friendship quality can act as a protective factor, potentially reducing NSSI behaviors in adolescents. By exploring this relationship, the study aims to offer valuable theoretical insights that can inform future intervention strategies to mitigate NSSI in this demographic.

As adolescents mature, their interpersonal relationships gradually shift from a primary focus on parents to an increasing reliance on peers (Brown and Larson, 2009). Friendship quality, which encompasses emotional support, companionship, and the degree of conflict within relationships, plays a crucial role in adolescents’ emotional well-being (Parker and Asher, 1993). During periods of emotional distress caused by adverse life events, adolescents may experience negative emotions such as loneliness, depression, and anxiety. However, high-quality friendships can mitigate these negative emotions, offering essential emotional relief (Kingery et al., 2011; Mounts, 2002). A study involving over 500 adolescents found a significant negative correlation between friendship quality and alexithymia (Peng and Huang, 2024), suggesting that friendship quality may moderate the detrimental effects of alexithymia. Moreover, the buffering effect of high-quality friendships can reduce the occurrence of externalizing behaviors (Burk and Laursen, 2005). Several studies also suggest that friendship quality may weaken the relationship between negative emotional symptoms and NSSI, preventing the onset of NSSI behaviors (Wang et al., 2021; Zhong et al., 2024).

The risk-buffering model provides a valuable framework for understanding the moderating role of friendship quality. According to this model, protective factors can alleviate the negative impact of risk factors (Cohen and Wills, 1985). Specifically, high-quality friendships can buffer the adverse effects of alexithymia on NSSI. Adolescents with lower levels of alexithymia and higher friendship quality are less likely to engage in NSSI than those with high alexithymia but low friendship quality. This may be due to the ability of high-quality friendships to reduce negative emotional attributions and foster the development of positive coping strategies (Li et al., 2022; Troop-Gordon et al., 2015). Thus, this study hypothesizes that friendship quality moderates the relationship between alexithymia and NSSI.

Research has shown that, as adolescents mature, they tend to disclose their feelings and thoughts more frequently to friends, and the level of support they receive from peers in their personal development increases. For adolescents, friendships serve as a vital source of social support, which can help reduce the occurrence of NSSI behaviors (Eggermont et al., 2021). High-quality friendships provide individuals with stable emotional support (e.g., the *security* subdimension) and practical problem-solving assistance (e.g., the *help* subdimension), among other benefits, which can effectively mitigate the risks associated with bullying victimization. Consequently, such friendships are more likely to play a protective role in reducing non-suicidal self-injury (NSSI), supporting the social support theory. This theory posits that strong social networks help individuals cope with psychological distress by offering multiple forms of support—including emotional support (e.g., reassurance and empathy), instrumental support (e.g., tangible aid in problem-solving), and informational support (e.g., advice and guidance)—thereby enhancing resilience against various mental health challenges (Bukowski et al., 1994; Garmezy, 1983; Kloo, 2025). A meta-analysis has found that adolescents who experience bullying are at a higher risk of NSSI compared to their non-bullied peers (Huang et al., 2022). However, not all adolescents who experience bullying exhibit adverse outcomes (Fitzpatrick and Bussey, 2014). One possible explanation for this discrepancy is the quality of adolescents’ interpersonal relationships, particularly the support provided by friends, which may mitigate the adverse effects of victimization, including NSSI (Kendrick et al., 2012). The social buffering model suggests that social support is a protective factor when individuals are exposed to risk factors, such as bullying (Cohen and Wills, 1985). As adolescents enter puberty, their emotional well-being becomes more reliant on peer support, with peer interactions and influences surpassing those of parents (Lyell, 2020). Consequently, friendship quality functions as a protective factor that moderates the adverse effects of risk factors, such as bullying, on adolescents (Cuadros and Berger, 2016). Despite these insights, no study has examined whether friendship quality moderates the relationship between students’ experiences of bullying and NSSI. Therefore, this study proposes the hypothesis that friendship quality moderates the relationship between students’ experiences of bullying and NSSI in adolescents.

## 2 Goal of the study

**Figure 1:**
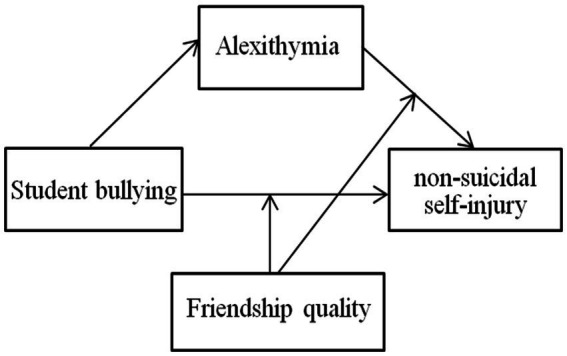
Conceptual model.

The objective of this study is to construct a moderated mediation model (see Figure 1) to thoroughly investigate the relationship between students’ experiences of bullying and NSSI. The following hypotheses are proposed:

*H1*: Students’ experiences of bullying have a direct and significant impact on NSSI behavior.

*H2*: Alexithymia mediates the relationship between students’ experiences of bullying and NSSI, thereby increasing the likelihood of NSSI.

*H3*: Friendship quality moderates the association between alexithymia and NSSI, thereby reducing the risk of NSSI.

*H4*: Friendship quality moderates the relationship between students’ experiences of bullying and NSSI, thereby alleviating the risk of NSSI.

**Figure 2:**
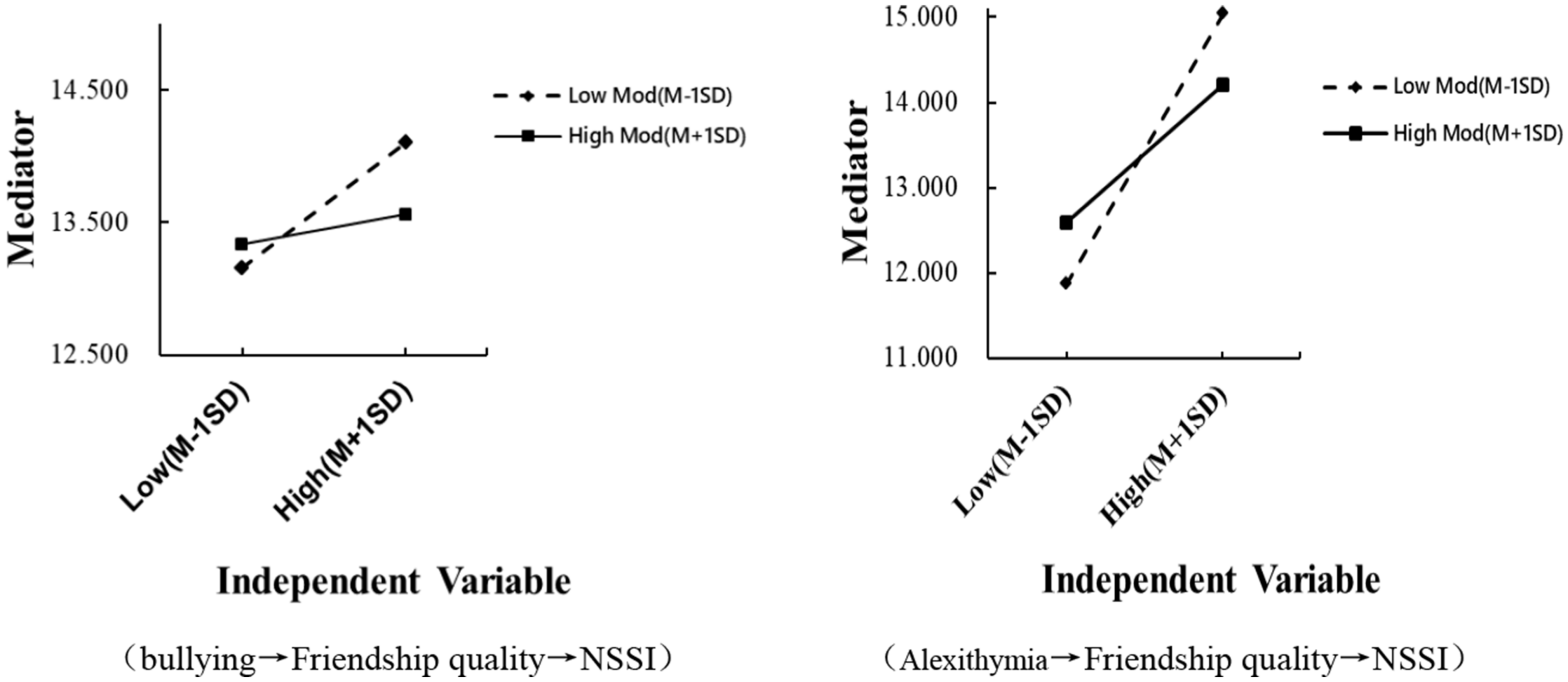
Friendship quality moderating the relationship between student bullying and non-suicidal self-injury.

This study, based on the impact of bullying and non-suicidal self-injury, approaches the issue from the perspective of emotional competence and uses friendship as a protective factor. It not only enriches the theoretical framework of the risk-buffering model by incorporating the quality of friendship as a buffering element but also explores the underlying mechanisms related to psychological issues, specifically alexithymia, as an intermediary (Figure 2). This practical approach offers a solid foundation for future, diversified, and precise interventions. Moreover, due to the complexity of the environment, which influences psychological development, and the limited research on rural adolescents with NSSI, this study holds greater urgency and social value compared to studies on urban adolescents, thus addressing the urbanization bias in existing research.

## 3 Research methodology

### 3.1 Participants

Adolescent students from six high schools in Tongliao City, Inner Mongolia, participated in a questionnaire survey. A total of 826 questionnaires were distributed, and 125 were excluded for insufficient responses, resulting in 701 valid participants, yielding an effective response rate of 84.7% [this response rate is meant to reflect valid participants/eligible participants (i.e., all students who attended the participating classrooms)]. Details of the valid participants are presented in Table 1. Participants were from several schools in Naihanqi of Tongliao City, with 550 participants (78.5%) from rural areas and 133 participants (19.0%) from urban areas. Among the participants, 187 (26.7%) held class leadership positions, while 493 (70.3%) did not. Also, 225 participants (32.1%) were only children, while 464 (66.2%) were not. In terms of family structure, 563 participants (80.3%) came from intact families, 67 (9.6%) came from single-parent families, and 56 (8.0%) came from blended families. Females constituted 51.16%, while males comprised 48.84% of all participants. As for grade levels, there were 63 participants (9.0%) in the first year of junior high school, with an average age of about 13; 118 participants (16.8%) in the second year, with an average age of 14; 144 participants (20.5%) in the third year, with an average age of 15; 150 participants (21.4%) in the first year of senior high school, with an average age of 16; 143 participants (20.4%) in the second year, with an average age of around 17; and 74 participants (10.6%) in the third year, with an average age of about 18. Data collection and entry for this study ranged from November 2023 to January 2024.

**Table 1:** Statistics on the number of boys and girls in each grade.

Grade	Male	Female	Total
First grade	27	36	63
Second grade	69	49	118
Third grade	72	72	144
Freshman year	80	70	150
Sophomore year	66	77	143
Senior year	24	50	74
Total	338	354	692

### 3.2 Inclusion and exclusion criteria

Inclusion Criteria: The participants must be between the ages of 12 and 18, properly understand the contents of the questionnaire, and be willing to cooperate with the survey. Additionally, consent must be obtained from both the participants and their guardians.

Exclusion Criteria: Exclusion criteria include the presence of neurological or other mental disorders, substance or alcohol dependency, conditions such as epilepsy, brain injury, or intellectual disabilities, recent exposure to significant stressful events, hearing, speech, or other communication impairments that affect everyday interaction, and an inability to cooperate with the surveyors.

### 3.3 Research tools

#### 3.3.1 Bullying questionnaire

This study used the Olweus Bullying Questionnaire, revised by Zhang and Wu (1999), to assess the frequency of bullying experiences over the past 3 months. The questionnaire consists of six items categorized into three dimensions: Verbal Bullying (e.g., “Some classmates give me ugly nicknames, curse me, or mock me” – 2 items), Physical Bullying (e.g., “Some classmates hit, kick, push, bump into, or threaten me” – 2 items), and Relational Bullying (e.g., “Some classmates spread rumors about me via phone or computer and try to make others dislike me” – 2 items). Respondents rate each item on a 5-point Likert scale, where 1 indicates “never happened,” 2 “only happened once or twice,” 3 “a few times a month,” 4 “about once a week,” and 5 “several times a week.” A higher total score indicates a higher level of bullying experienced. In this study, the factor-fit model for the bullying questionnaire demonstrated good reliability, with a Cronbach’s *α* coefficient of 0.81.

#### 3.3.2 Adolescent non-suicidal self-injury assessment questionnaire

The Adolescent Non-Suicidal Self-Injury Assessment Questionnaire, developed by Wan et al. (2018), was used to assess the occurrence of 12 types of self-injury behaviors over the past year. It comprises the Functional and Behavioral Questionnaires (Wan et al., 2018). The Behavioral Questionnaire contains 12 items, rated on a 5-point Likert scale. It is divided into two dimensions: “No Obvious Organizational Damage,” including behaviors such as intentionally scratching, pinching, or biting oneself (7 items), and “Obvious Organizational Damage,” including behaviors such as deliberately cutting oneself or using objects to rub the skin to cause bleeding or bruising (5 items). In this study, the factor-fit model for the self-injury questionnaire demonstrated good reliability, with a Cronbach’s *α* coefficient of 0.87.

#### 3.3.3 Toronto alexithymia scale (TAS-20)

The Toronto Alexithymia Scale (TAS-20), developed by Bagby et al. (1994) and revised by Yi et al. (2003), consists of 20 items designed to measure alexithymia across three dimensions: Difficulty in Identifying Feelings (e.g., “I often have trouble figuring out what kind of feelings I am experiencing” – 7 items).

Difficulty in Describing Feelings (e.g., “I can easily describe my feelings” – 5 items), and Externally-Oriented Thinking (e.g., “I prefer discussing others’ daily activities rather than their emotions” – 8 items). Respondents rate each item on a 5-point Likert scale, with higher scores indicating higher levels of alexithymia. In this study, the factor-fit model for the Toronto Alexithymia Scale showed good reliability, with a Cronbach’s *α* coefficient of 0.75.

#### 3.3.4 Friendship quality questionnaire

The Friendship Quality Questionnaire, revised by Zhou et al. (1998), was used to assess perceived social support in this study. This 38-item scale is divided into six dimensions: Affirmation and Care (e.g., “He/She tells me I am capable” – 3 items), Help and Guidance (e.g., “This friend often gives me advice on solving problems” – 3 items), Intimacy, Disclosure, and Communication (e.g., “We always discuss the problems we encounter together” – 3 items), Companionship and Entertainment (e.g., “When doing things, we always consider each other as a companion” – 3 items), Conflict Resolution Strategy (e.g., “When we have a dispute, it is easy for us to reconcile” – 3 items), and Conflict and Betrayal (e.g., “We often get angry with each other” – 3 items). The scale uses a 5-point Likert scale, where 1 represents “completely disagree” and 5 represents “completely agree.” The total score is calculated by summing the individual item scores, with higher scores indicating better friendship quality. In this study, the factor-fit model for the Friendship Quality Questionnaire exhibited excellent reliability, with a Cronbach’s α coefficient of 0.88.

### 3.4 Procedures

This study was approved by the Research Ethics Committee of Inner Mongolia University for Nationalities. After establishing contact with teachers from two middle schools in Naiman Banner, Tongliao City, the researcher introduced the purpose and content of the study. After acquiring their consent, the teachers in the local school used the paper version of the questionnaire to conduct the whole class test in their spare time, and the students completed the questionnaire anonymously. Written informed consent and assent forms were acquired from the participants and their legal guardians/relatives. Participation was voluntary, and confidentiality was guaranteed. The data collectors consisted of trained researchers who ensured the standardization of the data collection process. Completing the self-report questionnaire took approximately 30 min.

### 3.5 Data analysis

Initially, descriptive statistics and correlation analyses were conducted using SPSS 26.0. The next step involved performing regression analyses to assess potential violations of the underlying assumptions. A hypothesis was considered valid if the 95% confidence interval (CI) did not include zero. Subsequently, the mediation and moderated mediation models proposed by Hayes (2013) were applied using SPSS Models 4 and 15. A bootstrap sample of 5,000 was drawn from the data, and the 95% bootstrap confidence interval (CI) was calculated. Finally, gender and grade were included as covariates in the analysis.

## 4 Results

### 4.1 Descriptive statistics and correlation analysis

Table 2 presents the means, standard deviations, and correlation coefficients for the variables examined in this study. The analysis revealed significant correlations among all variable pairs: students’ experiences of bullying, alexithymia, friendship quality, and non-suicidal self-injury (NSSI). Notably, friendship quality was negatively correlated with NSSI, students’ experiences of bullying, and alexithymia. This suggests that higher friendship quality is associated with a lower likelihood of engaging in NSSI, while lower friendship quality is linked to an increased risk of bullying victimization. Additionally, individuals with higher friendship quality exhibited lower levels of alexithymia. Conversely, significant positive correlations were observed between the other variables. Specifically, more frequent experiences of school bullying were associated with more severe alexithymia symptoms, and a greater degree of bullying experiences was linked to more frequent engagement in NSSI behaviors. Similarly, higher levels of alexithymia were associated with more severe NSSI scores. These findings provide empirical support for the hypotheses tested in the subsequent analysis.

**Table 2:** Descriptive statistics and correlations between variables.

Variable	M ± SD	1	2	3	4	5	6
1. Gender	1.51 ± 0.50	-					
2. Grade	3.60 ± 1.48	0.08*	-				
3. Student bullying	6.81 ± 1.83	−0.12**	−0.03	-			
4. Alexithymia	55.55 ± 9.58	0.09*	0.09*	0.13**	-		
5. Friendship quality	64.01 ± 12.30	0.10**	0.05	−0.20**	−0.25**	-	
6. Non-suicidal self-injury	13.56 ± 3.78	−0.03	−0.08*	0.22**	0.31**	−0.12**	-

*n* = 701, M, mean; SD, standard deviation; *p < 0.05, **p < 0.01, ***p < 0.001.

### 4.2 Test of the mediating model of alexithymia

According to Table 3, an *F*-test was conducted to evaluate the model, and the results indicated model validity (*F* = 34.01, *p* = 0.000 < 0.001). The total regression coefficient for student bullying was 2.67 (*t* = 5.83, *p* = 0.000 < 0.001), suggesting that student bullying has a significant positive impact on non-suicidal self-injury behaviors.

**Table 3:** Regression analysis of student bullying and non-suicidal self-injury.

Predictors	Unstandardized coefficients		Standardized coefficients	*t*	*p*
	*B*	SE	Beta		
Constant	10.535	0.537	–	19.602	0.000
Student bullying	2.666	0.457	0.215	5.832	0.000
*R* ^2^			0.046		
Adjusted *R*^2^			0.045		
F	34.013***				
D-W	1.796				

*p < 0.05, **p < 0.01, ***p < 0.001.

The mediation model was tested using SPSS Process Model 4, with gender and grade as control variables. The analysis revealed a significant positive association between students’ experiences of bullying and NSSI (*β* = 2.11, *p* < 0.001) (Table 4). As shown in Table 4, when alexithymia was added to the model, a significant positive correlation between students’ experiences of bullying and alexithymia was observed (*β* = 4.43, *p* < 0.001), and alexithymia was also positively associated with NSSI (*β* = 0.12, *p* < 0.001). Furthermore, the Bootstrapping test results, presented in Table 5, indicated that the 95% confidence intervals for both the indirect effect of alexithymia and the direct effect of students’ experiences of bullying on NSSI did not include zero ([0.00, 1.23], [0.22, 0.94]). Meanwhile, the mediating effect was 0.53, accounting for 20.08% of the total effect. These findings suggest that alexithymia partially mediates the relationship between students’ experiences of bullying and NSSI. Collectively, these results provide empirical support for Hypotheses 1 and 2.

**Table 4:** Mediation model of alexithymia between student bullying and non-suicidal self-injury.

Predictors	Alexithymia			Non-suicidal self-injury		
	*β*	SE	95%CI	*β*	SE	95%CI
Constant	45.82***	2.03	[0.00, 41.83]	5.75***	1.01	[3.77, 7.72]
Gender	1.82*	0.72	[0.01, 0.40]	−0.17	0.27	[−0.71, 0.37]
Grade	0.57*	0.24	[0.09, 1.05]	−0.27**	0.09	[−0.45, −0.09]
Student bullying	4.43***	1.18	[2.12, 6.74]	2.11***	0.45	[1.23, 2.99]
Alexithymia				0.12***	0.01	[0.09, 0.15]
*R* ^2^	0.03			0.14		
*F*	8.15***			27.85***		

*n* = 692. Bootstrap sample size = 5,000. CI, confidence interval. β = standardized coefficient; *p < 0.05, **p < 0.01, ***p < 0.001.

**Table 5:** Bootstrapping indirect effect and 95% confidence interval (CI) for the mediation model.

Effect	Estimated effect	*SE*	95%*CI*	Ratio to total effect
Total effect	2.64	0.46	[0.00, 1.73]	
Direct effect	2.11	0.45	[0.00, 1.23]	79.92%
Indirect effect	0.53	0.18	[0.22, 0.94]	20.08%

*n* = 692. Bootstrap sample size = 5,000; CI = confidence interval.

### 4.3 Test of the moderating model of friendship quality

The moderated mediation model was tested using SPSS Process Model 15. As shown in Table 6, the interaction term between students’ experiences of bullying and friendship quality had a significant direct effect on non-suicidal self-injury (NSSI) (*β* = −0.07, *p* < 0.05), and the interaction term between alexithymia and friendship quality also significantly influenced NSSI (*β* = −0.003, *p* < 0.01).

**Table 6:** Moderated mediation model of friendship quality between student bullying and non-suicidal self-injury through Alexithymia.

Predictors	Alexithymia			non-suicidal self-injury		
	*β*	SE	95%CI	*β*	SE	95%CI
Constant	−4.80***	1.39	[−7.54, −2.06]	14.53***	0.53	[13.49, 15.57]
Gender	1.82*	0.72	[0.40, 3.24]	−0.11	0.27	[−0.64, 0.43]
Grade	0.57*	0.24	[0.09, 1.05]	−0.26**	0.09	[−0.44, −0.08]
Student bullying	4.43***	1.18	[2.12, 6.74]	1.33*	0.54	[0.26, 2.40]
Alexithymia				0.13***	0.01	[0.10, 0.15]
Friendship quality				−0.002	0.01	[−0.03, 0.02]
Student bullying × Friendship quality				−0.07*	0.03	[−0.13, −0.01]
Alexithymia × Friendship quality				−0.003**	0.00	[−0.01, −0.00]
*R^2^*	0.03			0.16		
*F*	8.15***			18.87***		

*n* = 692. Bootstrap sample size = 5,000. CI = confidence interval. β = standardized coefficient; *p < 0.05, **p < 0.01, ***p < 0.001.

A simple slope analysis was conducted to explore the nature of these interactions. Participants were divided into two groups based on the level of moderation (friendship quality): low (M – SD) and high (M + SD). Among individuals with low friendship quality, a positive correlation between alexithymia and NSSI was observed (simple slope = 0.17, *p* < 0.001). In contrast, although the association remained significant for individuals with high friendship quality, it was weaker (simple slope = 0.08, *p* < 0.001). These findings suggest that higher levels of friendship quality attenuate the relationship between alexithymia and NSSI. These results support Hypothesis 3, confirming that friendship quality moderates the relationship between alexithymia and NSSI.

Similarly, when participants were divided into two groups based on friendship quality (low vs. high), a positive correlation between students’ experiences of bullying and NSSI was observed among individuals with low friendship quality (simple slope = 2.15, *p* < 0.001). However, for individuals with high friendship quality, this correlation, while still significant, was weaker (simple slope = 0.51, *p* < 0.001). These results indicate that higher levels of friendship quality diminish the strength of the relationship between students’ experiences of bullying and NSSI. Collectively, these findings support Hypothesis 4, confirming that friendship quality moderates the relationship between students’ experiences of bullying and NSSI.

## 5 Discussion

### 5.1 Bullying and non-suicidal self-injury

This study demonstrates that students’ experiences of bullying are a direct predictor of NSSI behaviors, aligning with previous research indicating that students who experience bullying are at a heightened risk of engaging in NSSI (Li et al., 2020). When individuals encounter stressful situations, they cannot resolve or adapt. They may resort to maladaptive coping mechanisms to alleviate the emotional distress caused by these experiences. According to the general stress model, individuals exposed to environmental stressors like bullying and unable to modify their behaviors to cope with these stressors experience a subjective sense of oppression. This heightened distress increases their likelihood of engaging in extreme behaviors, such as NSSI or avoidance, to manage or escape the negative emotions induced by stress (Van Geel et al., 2015). Consequently, adolescents who experience bullying are more likely to develop NSSI behaviors compared to their non-bullied peers.

### 5.2 The mediating role of alexithymia in bullying and non-suicidal relationships

The findings of this study indicate that students’ experiences of bullying indirectly affect adolescents’ NSSI behaviors through alexithymia, thereby increasing the risk of NSSI. This result corroborates the work of Guo et al. (2024), who found that alexithymia mediates the relationship between bullying victimization and adolescent NSSI. Specifically, bullied students tend to exhibit higher levels of alexithymia compared to those who are not, and individuals with higher alexithymia levels are more likely to engage in NSSI behaviors (Guo et al., 2024; Wen et al., 2024).

On the one hand, victims of bullying often experience fragmented memories and a narrowed focus of attention (van der Kolk and Fisler, 1995), making it difficult for them to fully utilize their cognitive resources to process emotional states and physiological responses (Lischke et al., 2022). This impairment in emotional processing hinders their ability to recognize and express their own and others’ emotions, further preventing them from regulating traumatic feelings and exacerbating emotional dysregulation (Cowie and Berdondini, 2002). As a result, bullied students are more likely to develop alexithymia. On the other hand, the chronic exposure to trauma, such as bullying, generates persistent negative emotions, which adolescents may attempt to manage through maladaptive coping mechanisms like NSSI. This pattern of negative reinforcement exacerbates the cycle of emotional dysregulation. Therefore, individuals with alexithymia are more likely to engage in NSSI behaviors as a way to cope with emotional distress (Hasking, 2016). Empirical studies also support the notion that alexithymia is a direct predictor of NSSI (Zhang et al., 2023), making it one of the most significant predictors of NSSI (Cameron et al., 2014; Taylor and Bagby, 2004; Swiller, 1988).

Furthermore, these findings align with the social mechanism of alexithymia, which suggests that negative social experiences in childhood can disrupt an individual’s psychological well-being and reduce their capacity for social interaction (Honkalampi et al., 2004). Consequently, individuals who have experienced bullying may have impaired emotional regulation and self-evaluation processes, which contribute to alexithymia and significantly predict the risk of NSSI in adolescents (Guo et al., 2024). Severe alexithymia was associated with exacerbated NSSI following bullying, though this effect may be buffered by high friendship quality in certain individuals.

### 5.3 Moderating effect of friendship quality

Finally, the study found that friendship quality moderates the relationship between alexithymia and NSSI.

Specifically, individuals with higher-quality friendships exhibited lower NSSI scores than those with lower-quality friendships and higher levels of alexithymia. These findings suggest that friendship quality may act as a protective factor against alexithymia. Previous research similarly demonstrated that friendship quality moderates the impact of negative emotions on NSSI (Wang et al., 2021), in line with the Social Buffering Model. According to this model, friendship quality, as a form of social support, can mitigate the negative effects of adverse experiences (Cohen and Wills, 1985). Therefore, when adolescents experience higher levels of friendship quality, it can alleviate negative emotions and reduce the likelihood of maladaptive behaviors, including NSSI (Kim, 2015; Sinanan and Gomes, 2020; Woods et al., 2009). Empirical studies also support this notion, showing that lower-quality friendships are associated with more pronounced alexithymia symptoms (Peng and Huang, 2024), while individuals with higher-quality friendships tend to engage in fewer NSSI behaviors (Eggermont et al., 2021). Moreover, NSSI is often considered an unhealthy emotion regulation strategy and maladaptive coping behavior (Klonsky, 2009). Adolescents with high-quality friendships are more likely to manage psychological distress through social support and communication rather than resorting to NSSI.

Additionally, the study found that friendship quality also moderates the relationship between students’ experiences of bullying and NSSI. Specifically, individuals with higher-quality friendships exhibited lower NSSI scores than those with lower-quality friendships. These findings are consistent with those of Wang et al. (2021), who showed that friendship quality moderated the relationship between cyberbullying and NSSI. Most studies consistently indicate that friendship quality moderates the relationship between adverse experiences and severe outcomes (Bernasco et al., 2022; Li et al., 2023; Li et al., 2022). High-quality friendships are characterized by low levels of conflict/arguments and high levels of positive features, including prosocial behaviors, loyalty, intimacy, and self-esteem support. Notably, the constructive subdimensions of such friendships can effectively mitigate the severe consequences associated with bullying victimization (Kloo, 2025). Furthermore, the conclusions of this study align with the Social Buffering Model, which suggests that individuals with higher-quality friendships are better equipped to cope with challenges through emotional support and problem-solving strategies derived from interpersonal interactions. This helps mitigate the adverse effects of bullying. Therefore, in similar risk environments, individuals with high-quality friendships are less likely to engage in harmful behaviors (Cohen and Wills, 1985).

Moreover, according to the General Strain Theory, recent exposure to other major stressful events (included in the exclusion criteria) may also have a potential impact on NSSI. Additionally, studies have shown that substance or alcohol dependency is associated with NSSI behaviors, while speech or other communication impairments could significantly confound data analysis. Therefore, these factors were deemed appropriate exclusion criteria to enhance the robustness of the study findings.

### 5.4 Implications for theory, research, and practice

This study contributes to the social mechanism perspective of alexithymia, providing valuable insights into its role as a significant predictor of NSSI in the context of student bullying. Specifically, the results show that students’ experiences of bullying indirectly affect adolescent NSSI through the mediating role of alexithymia. Furthermore, our findings deepen the understanding of general stress theory, the social mechanism perspective of alexithymia, the cognitive-emotional model of NSSI, and social buffering theory. They also highlight the moderating role of friendship quality in both the relationship between students’ experiences of bullying and NSSI and the relationship between alexithymia and NSSI.

Firstly, Given that students’ experiences of bullying are significant predictors of non-suicidal self-injury (NSSI) behavior, it is essential for schools to address bullying incidents swiftly and per established protocols. Such incidents should be treated with the highest priority, ensuring prompt intervention. In cases where necessary, parental involvement should be sought to provide guidance and support, facilitating a timely and effective resolution. On the other hand, in the absence of bullying incidents, schools should conduct regular seminars and awareness campaigns for students and parents to promote self-protection, raise parental awareness, and establish and implement robust prevention and intervention policies (Guo et al., 2024). These policies should include the development of a comprehensive monitoring and disciplinary system (Li et al., 2020), fostering close collaboration between school staff and parents to form a supportive school-family partnership. This partnership should focus on closely monitoring students’ psychological well-being and behavioral indicators, ensuring the early identification of potential bullying victims and the implementation of targeted interventions. By addressing bullying early, these measures will help reduce bullying incidents and provide a foundation for lowering the incidence of NSSI. More importantly, the school-family partnership should actively involve students, encouraging them to develop effective coping strategies. Influenced by a supportive campus environment and related activities, students can enhance their ability to manage stress, navigate challenges, and learn to protect themselves and their peers from harm.

Secondly, the present study highlights the significant role of alexithymia in the relationship between student students’ experiences of bullying and non-suicidal self-injury. Given this, parents and educators need to promote the development of emotional regulation strategies and social skills in students, particularly those exhibiting symptoms of alexithymia, as they may face greater challenges in managing their emotional responses to adverse experiences. Schools and families can facilitate this process by organizing group activities, such as team-based games, to enhance students’ ability to recognize, express, and regulate their emotions (Guo et al., 2024; Zhang et al., 2023). Using appropriate language, these activities should encourage students to articulate their inner experiences and feelings openly. Such interventions may help alleviate the negative symptoms commonly associated with alexithymia. In addition, students should be encouraged to engage in regular self-reflection and external communication to improve their emotional awareness and coping skills. Parents, in turn, must prioritize their children’s emotional development by fostering an environment conducive to emotional expression and communication. They should stay attuned to their children’s emotional states and guide them in developing effective coping strategies to enhance emotional resilience and well-being.

Finally, considering the pivotal role of friendship quality in moderating the relationship between students’ experiences of bullying and NSSI, schools need to create opportunities for positive peer interactions at key moments, fostering mutual support and expanding students’ social networks. For example, schools could organize friendship-building activities across different classes to establish and strengthen positive friendships among middle school students. These initiatives would promote the formation of supportive social connections and enhance the overall quality of their relationships. Furthermore, schools should periodically rearrange class groups to increase students’ opportunities to meet new peers and form diverse social connections (Cuadros and Berger, 2016). Such interactions help students develop essential social skills and significantly improve the quality of their friendships. In parallel, students should be actively encouraged to develop a deeper understanding of friendship dynamics and social skills, focusing on cultivating interpersonal abilities that improve the quality of their relationships. Students can build positive, supportive friendships that promote mutual growth by intentionally fostering these skills. It is also important to note that the quality of students’ friendships can be influenced by family dynamics and relational patterns within the home environment. Consequently, parents should actively nurture their children’s social skills, create a supportive atmosphere that encourages friendship-building, and offer emotional support, all of which contribute to developing their children’s social competence and emotional well-being.

### 5.5 Limitations and future recommendations

Although this study makes significant contributions by exploring key issues and uncovering the underlying patterns of essential phenomena, it also reveals certain limitations that should be addressed in future research. These limitations provide opportunities to refine the findings and enhance their robustness. First, the study’s participant pool was restricted to rural middle school students, which may limit the generalizability of the results. Future research should, therefore, consider including urban middle school students in the sample and establish a control group for comparative analysis. Such an approach would broaden the findings’ scope and improve the conclusions’ comprehensiveness and validity.

Second, this study utilized a cross-sectional design, which, while effective for examining relationships between variables at a single point in time, does not capture dynamic changes or long-term developmental trends. Future studies should incorporate longitudinal tracking methods to better understand and validate the complex relationships between variables. This would involve conducting long-term follow-up observations and data analysis to capture the evolving nature of the variables over time, thereby enhancing the reliability and stability of the findings. Finally, this study primarily focused on the theoretical discussion of mediating and moderating effects, providing valuable conceptual insights for the field. However, its practical applications and implications for real-world interventions remain limited. Future research should expand its scope by incorporating intervention studies to address this gap. By designing and implementing targeted intervention strategies, future studies could assess the feasibility and impact of the theoretical findings, providing more concrete and actionable guidance for real-life applications. Moreover, such research would offer valuable lessons and insights to inform future intervention efforts. Finally, negative friendship traits, including conflict-ridden relationships, can lead to various adverse outcomes, thereby increasing the risk of victimization. However, this study lacks an in-depth examination of the relationship between specific subdimensions of friendship quality and the dependent variables. Future research exploring these associations could yield more profound insights.

## 6 Conclusion

Students’ experiences of bullying not only directly influence NSSI behavior but also indirectly affect it through alexithymia. Additionally, friendship quality moderates both the relationship between students’ experiences of bullying and NSSI, as well as the association between alexithymia and NSSI. These findings suggest potential intervention strategies to reduce the prevalence of NSSI among students and provide valuable insights into addressing NSSI behaviors. Furthermore, they highlight important avenues for future research, particularly concerning the underlying mechanisms of NSSI.

## Data Availability

The datasets presented in this study can be found in online repositories. The names of the repository/repositories and accession number(s) can be found here: https://doi.org/10.6084/m9.figshare.29964440. Further inquiries can be directed to the corresponding author.
